# Predicting Future Blood Demand from Thalassemia Major Patients in Hong Kong

**DOI:** 10.1371/journal.pone.0081846

**Published:** 2013-12-11

**Authors:** Eric H. Y. Lau, Xiu-Qing He, Cheuk-Kwong Lee, Joseph T. Wu

**Affiliations:** 1 The University of Hong Kong, Pokfulam, Hong Kong Special Administrative Region, China; 2 Hong Kong Red Cross Blood Transfusion Service, 15 King’s Park Rise, Hong Kong Special Administrative Region, China; Cardiff University, United Kingdom

## Abstract

**Background:**

In Hong Kong, thalassemia major (TM) patients utilized up to 9.5% of blood supply in 2009. For long-term management of blood supply, we predicted the future blood demand of TM patients for the next 10 years.

**Methods and Results:**

Annual individual transfusion data in 2005–2009 and demographic information of 381 TM patients were obtained from the Hong Kong Red Cross Blood Transfusion Service database. A generalized estimating equation (GEE) model was fitted to establish the potential relations of blood demand with age, sex, body weight, year of transfusion and splenectomy, accounted for within-patient correlation. The fitted model was used to predict future blood demand for the existing patients by accounting for expected change in body weight and mortality rate. We also predicted the number of new cases in the future based on age- and sex-specific TM incidence and official population projections. Future blood demand was predicted by combining blood demand from the existing and new patients. Female (RR = 0.94, p = 0.006) and history of splenectomy (RR = 0.85, p<0.001) were significantly associated with lower blood demand, while age and weight had an inverted U-shape relation with maximal blood demand at around 24 years of age and 71.8 kg, respectively. We predicted that the total blood demand would increase 0.81% annually from 13,459 units in 2009 to 15,183 units in 2024, with new TM cases accounting for 31.7% of the overall blood demand in 2024.

**Conclusions:**

Our results showed that future annual blood demand from TM patients would steadily increase in the next 10 years. Reducing incidence of TM cases in the future (by improving public education, antenatal care, prenatal diagnosis) and minimizing blood use among existing TM cases (e.g. with hemopoietic stem cell transplantation) can help relieve the burden on management of future blood demand.

## Introduction

Blood demand has been increasing all over the world. Ageing of the population worldwide [1] together with higher cancer incidence in advanced ages is a major reason for increased blood utilization [2]. Life expectancy of both healthy and diseased people are expected to increase with continual advances in medical care and technologies. However, as more people survive longer, they are more likely to develop chronic illnesses and degenerative problems, thereby increasing blood demand. Although these patients may not be cured, medical management and blood transfusion allow them to survive longer (with some achieving close to normal life expectancy) but at the expense of large amount of blood use.

Hong Kong has a population of about 7 million with an annual blood supply of 226,718 units in the financial year 2010/11 [3]. All blood donation services, from collection to distribution, are managed by the Hong Kong Red Cross Blood Transfusion Service which is part of the Hong Kong Hospital Authority. Over the years, Thalassemia Major (TM) patients have been shown to use increasingly more blood and with less than 400 cases, they utilize up to 9.5% of annual blood supply in Hong Kong [4]. With good antenatal care, the number of new TM cases is not expected to increase significantly. However, with continual advances in medicine, particularly blood transfusion and iron chelating therapy, the survival of TM patients has been and will be substantially prolonged, as similarly observed among those patients with aplastic anemia [5]. This will increase not only the associated health care cost, but more importantly, have direct implication in the provision of blood supply. Hong Kong has a rapidly ageing population which requires more blood transfusion and also ageing of first time blood donors at the same time [6]. A large effort has been invested in the recruitment of new donors as well as retention of existing donors to maintain stable and sufficient blood supply [7]. Hence, prediction of blood demand from this group is important for long-term management of blood supply. In this paper, we aimed to predict the blood demand of TM patients in Hong Kong in the next 10 years in order to allow better planning of blood services. Methods for prediction of blood demand have been employed for emergency medicine and surgery [8], [9], [10] or at a population level [11], [12], but no studies have looked into future blood demand from patients who require chronic transfusion. Here we proposed a practical statistical model which takes into account the impact of future growth in body weight of TM patients and nonlinear effects of age on blood demand from this group in the future.

## Methods

We retrospectively examined transfusion request records of all TM patients who required chronic blood transfusion in public hospitals of Hong Kong between 1 January 2005 and 31 December 2009. All public hospitals in Hong Kong are managed by the Hong Kong Hospital Authority and these records accounted for essentially all blood demands from TM patients in Hong Kong during this period. Annual blood demand for each patient was recorded along with patient information on sex, age, body weight and history of splenectomy by 2009. Survival rate of TM patients has improved significantly during the last decade because of recent medical advances and patient management [13]. As such, only the death counts in 2005–2009 were used to predict future mortality of TM patients though data from earlier periods were available [4].

### Prediction of Blood Demand from Existing Patients

We fitted a Poisson generalized estimating equations (GEE) model [14] for above-mentioned blood demand (the outcome variable) to establish the potential relation of blood demand from TM patients with age, sex, body weight, years of transfusion and history of splenectomy. All patients who had irregular blood transfusion (e.g. those who submitted transfusion request every other year) and incomplete records were excluded. Blood transfusion records for patients younger than 12 months were not used in the prediction model because TM patients typically start requiring blood transfusion therapy any time between 6 and 12 months of age. Past body weights in 2005–2008 were imputed based on the US CDC growth curve (details below).

The Poisson GEE model was chosen in order to account for the correlation between different episodes of blood demand from the same TM patient and potential overdispersion of the data by using the robust sandwich estimator for the variances [15]. We selected the potential predictors and appropriate correlation structure by the leave-one-out cross-validation method [16], where the predictive performance of the model was evaluated by iteratively fitting the model using all but one observations, and the predicted values were compared against the observations being left out. In the base model we included known important predictors of blood demand such as age, sex, body weight and history of splenectomy [17], [18], while other model variants including combinations of predictors such as years of transfusion and quadratic terms of weight and age were compared. We aim at developing a simple and practical model to predict long term blood at the population level including most important predictors [17], [18] and potentially important interaction effects particularly with age or weight. Mean absolute prediction error (MAE) between predicted and observed blood demand in the validation dataset was used to assess the predictive performance of the models.

The selected model was used to predict future blood demand for each patient accounting for expected changes in body weight in the future among adolescents and children. More specifically, we transformed the weight of each TM patient into z-scores using the LMS method [19], and used the CDC growth curve [20] to project future weight assuming that the z-score of each patient remained constant. For patients with missing body weight, we imputed their expected weight using the mean z-score in the same age group. We assumed body growth was negligible after the age of 20. Blood demands in 2010–2024 of the existing TM patients were predicted based on the fitted model and projected weights.

### Prediction of Blood Demand from New Cases

Future blood demand was calculated as the sum of demands from both existing TM patients and new cases of TM patients in the future. We estimated the number of new TM cases by assuming that age- and sex- specific incidence rate of TM in 2010–2024 would be the same as in the 2006–2009 data. These incidence rates were then applied to the projected population in 2010–2024 made by the Census and Statistics Department [21]. We stratified patients by sex and by age groups 0–4, 5–9, 10–19 and 20–59 years respectively. Assuming the new TM cases had similar blood demand characteristics as existing cases, we predicted their future blood demand based on the fitted Poisson GEE model using the group means for the explanatory variables. Prediction intervals were calculated by the delta method [22] which accounted for uncertainties in the estimated blood demand from the GEE model and the forecast error in the population projections.

We also calculated the annual mortality rate of TM patients based on the death counts in 2005–2009. As patients younger than 20 years had high survival rate and there are limited recent data on the long-term survival rate [23], [24], we applied a constant mortality rate to patients aged 20 years or above and adjusted the predicted blood demand by deducting blood usage from the death cases. All analyses were implemented in R version 2.15.2 [25]. Details of the prediction methods were described in Text S1.

## Results

There were a total of 381 TM patients in the dataset with 189 (49.6%) males and 192 (50.4%) females. Age ranged from 3 months to 56 years with a median of 23 years. Mean body weight was 46.5 kg and 138 (36.2%) of the patients had undergone splenectomy.

338 patients were included in the Poisson GEE model. 31 patients (8.1%) and 12 patients (3.1%) were excluded due to irregular blood transfusion records and missing records of body weights, respectively. Mean annual blood demand of these patients who required regular transfusion was 38.7 units. We found that there was little multicollinearity between predictors as all variance inflation factors (VIF) were below 3 for all predictors. Among all the model variants, the best model included sex, quadratic effects of age and weight, year of transfusion and history of splenectomy as predictors for blood demand, with an independent correlation structure (zero within-patient correlation between blood demand at different years), with a minimum MAE of 5.787 (Table 1). Considering that models including quadratic effects of age and weights performed markedly better but those models including year of transfusion only negligibly improved the predictive power, we excluded the predictor year of transfusion in our final predictive model. For the same reason, we did not include interaction terms which only improved the predictive power slightly to a MAE of 5.767 (Table S1).

**Table 1:** Mean absolute prediction error of the predicted blood demand under different model variants by leave-one-out cross-validation.

	Correlation structure	
Model variant	Independent	Exchangeable
Base model*	6.814	6.827
+ age^2^	6.002	6.040
+ weight^2^	6.058	6.112
+ year of transfusion	6.814	6.821
+ age^2^, weight^2^	5.792	5.830
+ age^2^, year of transfusion	5.997	6.026
+ weight^2^, year of transfusion	6.063	6.103
+ age^2^, weight^2^, year of transfusion	5.787	5.810

include predictors sex, history of splenectomy, linear effects of age and weight only.

**Figure 1:**
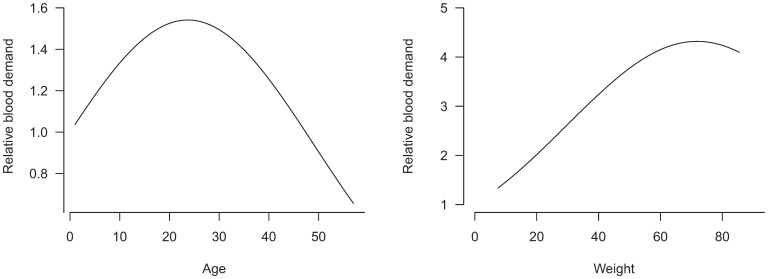
Estimated effects of age and weight on blood demand, based on the fitted Poisson generalized estimating equations model.

Table 2 shows the estimated effect of the predictors on blood demand. Age and weight were found to have an inverted U-shape relation with blood demand with a maximum demand at age 23.7 years and 71.8 kg respectively (Figure 1). Blood demand also increased with body weight but at a slower rate for those with higher body weight. Females and those who had undergone splenectomy had 5.6% and 15.4% less blood demand. No significant time trend in blood transfusion demand was found.

**Table 2:** Associations between blood demand and age, weight, sex and history of splenectomy using Poisson generalized estimating equations model, with the associated 95% CI, and p-values.

Variables	Relative blood demand*	95% CI†	P-value
Age	1.04	(1.02, 1.05)	<0.001
Age^2^	0.9992	(0.9989, 0.9995)	<0.001
Weight	1.04	(1.03, 1.05)	<0.001
Weight^2^	0.9997	(0.9996, 0.9998)	<0.001
Female	0.94	(0.91, 0.98)	0.006
Splenectomy	0.85	(0.81, 0.89)	<0.001

CI confidence Interval.

mutually adjusted for age, weight, sex and history of splenectomy.

% CIs. the estimated scale parameter was 1.71 which indicated moderate overdispersion. The sandwich estimator for the standard errors were used to construct the 95

**Figure 2:**
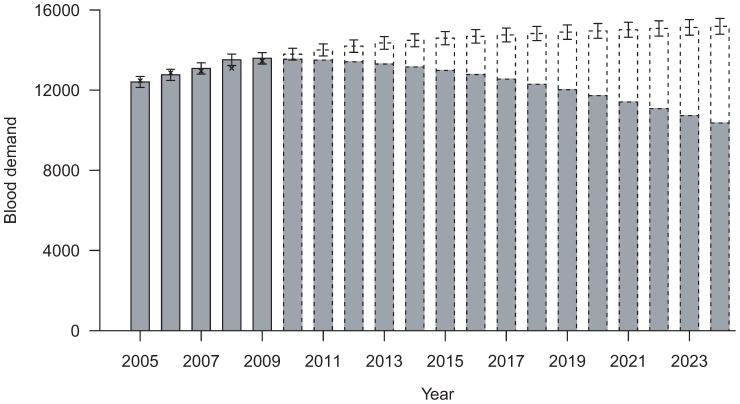
Predicted blood demand and prediction intervals of Thalamessia Major patients, 2010–2024. Crosses show the actual blood demand in 2005–2009.

Total blood demand in existing TM patients increased 1.9% annually from 12,469 units in 2005 to 13459 units in 2009. Based on the fitted GEE model, we predicted that blood demand from existing TM patients would decrease to 10,363 units (95% prediction interval PI = 10,067 to 10,658) in 2024 (Figure 2). We estimated that the annual incidence of TM cases requiring chronic transfusion was 0.13/100,000 (95% CI = 0.06 to 0.24/100,000), which translated to about 9–10 new TM cases every year in year 2010–2024. The annual mortality rate was estimated to be 0.96% (95% CI = 0.78% to 1.13%). By including these new patients and accounted for the death cases, the total blood demand was predicted to increase 0.81% annually to 15,183 units (95% PI = 14,787 to 15,579) in 2024 with new cases accounting for 31.7% of the overall TM blood demand in 2024. As sensitivity analyses, assuming the mortality rate in TM patients reduced linearly by a further 40% or 80% in the next 10 years [4], the total blood demand was predicted to increase 0.95% or 1.21% annually to 15,656 units (PI = 15,261 to 16,052) or 16,129 units (95% PI = 15,734 to 16,525) respectively in 2024.

## Discussion

### Future Blood Demand by Thalassemia Major Patients

By considering important factors for predicting blood demand, in particular, expected changes in body weight of TM patients and age as a proxy of physical activity, we provided a realistic prediction of blood demand of TM patients in Hong Kong in the next 10 years. We predicted that future demand of blood transfusion from TM patients would increase at a rate of 0.81% annually, lower than the rate of 1.9% in 2005–2009. The annual population growth in Hong Kong for the same period has been projected to be 0.9% [21]. Our prediction took into account changes in demographic structure and expected new cases of TM patients requiring chronic blood transfusion. Currently, the combined effect of low fertility rate in Hong Kong (total fertility rate = 1.1) [26] and better survival of TM patients [4] results in a slower rate of increase in blood demand from these patients. Our predicted annual increase of blood demand from TM patients is lower than the expected increase of 2–3% in overall blood demand, even with significant improvement in survival [27]. However, with moderate improvement in the survival of TM patients, the growth of overall blood demand is expected to rise faster than population growth, which suggests that management of blood demand from TM patients is still important.

In the last decade, there has been a rapid increase in the proportion of births by women from mainland China (from 15.9% in 2001 to 46.1% in 2010), many of whom came from Guangdong province [28]. Hong Kong and Guangdong province have similar prevalence of beta-thalassemia carriage [29], [30], though regular transfusion therapy may not be always available for TM patients in Guangdong [31]. More recently, there has been upsurges of non-local obstetric patients who are not eligible for obstetric services at government subsidized rate and have had late or no antenatal screening before they gave birth in Hong Kong [32]. These patients are associated with late presentations of blood disease such as fetal haemoglobin Bart’s disease which may require intrauterine blood transfusion [33]. The children have full residency rights to enjoy medical services in Hong Kong and may disproportionately increase patients who need chronic transfusion therapy in the future.

Our proposed model utilized commonly available sources such as basic patient information and official population projection to predict future blood demand from TM patients, which can be easily applied to other populations with minor modification. In Hong Kong, TM incidence is likely to be stable due to well established antenatal care program and our results showed that blood transfusion from new TM cases will not increase total blood demand rapidly. In populations with increasing antenatal care coverage, the expected reduction in TM incidence should be accounted for in the prediction model. Potential benefits of improving antenatal coverage on blood usage can be assessed for better distribution and long-term management of blood demand.

Though our study has compared different model variants for more accurate prediction of blood demand in TM patients, there are several limitations worth noting. We showed that inclusion of the nonlinear effects of age and weight significantly improved the prediction of blood demand though our dataset covered only 5 years of data and did not allow comparison of prediction accuracy of different models over a longer period. Another limitation was that the time of splenectomy was not available in the dataset. While splenectomy is negatively associated with blood demand, our model assumed a constant status and might have underestimated the effect of splenectomy on the reduction of blood demand. However, in our dataset there were less than 40% of the TM patients who have ever undergone splenectomy, it has been estimated that only less than 10% of TM patients had undergone splenectomy in 2006–2009. As such, its impact on our estimates should be limited. Although the cross-sectional nature of our analysis does not provide strong evidence to establish a causal relation between blood demand reduction and splenectomy, other studies have demonstrated a similar relation [17], [18]. While projected changes in blood demand from existing TM patients has been considered in our analysis, our prediction of blood demand from new TM cases would be sensitive to changes in TM incidence or demographic trends not reflected in the population projection made by the Census and Statistics Department in Hong Kong, such as new trends in fertility rate or cross-border deliveries, or breakthrough in the treatment of TM which may reduce transfusion requirement. Also, for practical reasons, detailed clinical data were not used in the planning of blood demand and the model may leave out potential confounders.

In conclusion, our results show that future annual blood demand from TM patients who require regular blood transfusion will increase by 12.8% from 13,459 units in 2009 to 15,183 units in 2024. Reducing incidence of new TM cases through public education, antenatal care, prenatal diagnosis, along with minimizing blood use in existing cases with hemopoietic stem cell transplantation, may help improve long-term management of future blood demand in Hong Kong.

## Supporting Information

Table S1 **Mean absolute prediction error of the predicted blood demand under different model variants with interaction terms and independent correlation structure, by leave-one-out cross-validation.**
(DOCX)

Text S1 **Description of the prediction method for blood demand from Thalassemia patients.**
(DOC)
