# Supplementary material for: Predicting Future Blood Demand from Thalassemia Major Patients in Hong Kong
Source: PLoS One. 2013 Dec 11;8(12):e81846. doi: 10.1371/journal.pone.0081846 (PMC3859512; doi:10.1371/journal.pone.0081846)
Supplement: Table S1 — Mean absolute prediction error of the predicted blood demand under different model variants with interaction terms and independent correlation structure, by leave-one-out cross-validation. (DOCX) [file pone.0081846.s001.docx]

Table S1. Mean absolute prediction error of the predicted blood demand under different model variants with interaction terms and independent correlation structure, by leave-one-out cross-validation.

| Model variant |  | Mean absolute prediction error |
| --- | --- | --- |
| Base model^*^ |  | 5.792 |
| + female × age, female × age^2^ (A) |  | 5.800 |
| + female × weight, female × weight^2^ (B) |  | 5.790 |
| + female × history of splenectomy (C) |  | 5.793 |
| + history of splenectomy × weight, history of splenectomy × weight^2^ (D) |  | 5.769 |
| + (A), (B) |  | 5.808 |
| + (A), (C) |  | 5.807 |
| + (A), (D) |  | 5.767 |
| + (B), (C) |  | 5.792 |
| + (B), (D) |  | 5.770 |
| + (C), (D) |  | 5.773 |
| + (A), (B), (C) |  | 5.817 |
| + (A), (B), (D) |  | 5.775 |
| + (A), (C), (D) |  | 6.018 |
| + (B), (C), (D) |  | 5.773 |
| + (A), (B), (C), (D) |  | 5.784 |

^*^include predictors sex, history of splenectomy, quadratic effects of age and weight only.
