# Supplementary material for: Predicting Future Blood Demand from Thalassemia Major Patients in Hong Kong
Source: PLoS One. 2013 Dec 11;8(12):e81846. doi: 10.1371/journal.pone.0081846 (PMC3859512; doi:10.1371/journal.pone.0081846)
Supplement: Text S1 — Description of the prediction method for blood demand from Thalassemia patients. (DOC) [file pone.0081846.s002.doc]

**Text S1. Description of the prediction method for blood demand from Thalassemia patients**

Prediction of blood demand from existing Thalassemia patients

We fitted a Poisson generalized estimating equations (GEE) model for the outcome variable annual blood demand to establish its potential relation with age, sex, body weight, years of transfusion and history of splenectomy, based the data from 2005 to 2009. All patients who had irregular blood transfusion (e.g. submitted transfusion request every other year) and incomplete record were excluded. Blood transfusion records for patients younger than 12 months were excluded because Thalassemia (TM) patients typically start requiring blood transfusion therapy any time between 6 and 12 months of age.

The GEE model was specified by:


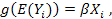


where *g* is the log-link function, *X_i_* included explanatory variables such as age, sex, body weight, years of transfusion, history of splenectomy and the quadratic terms for age and body weights, *β* is the coefficient for the explanatory variables. We allowed for independent or exchangeable correlation structure within the same patient. The most parsimonious model was chosen with the lowest root mean squared error in the leave-one-out cross validation [1].

An independent correlation structure was shown to be better in describing the within-patient correlation between blood demands in different years. The final model included explanatory variables age, sex, body weight, history of splenectomy and quadratic effect of age and body weight. It was used to predict future blood demand from existing TM patients
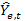
*t* years after 2009, by imputing corresponding ages and expected body weights in the subsequent years, given by:


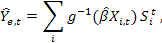


where *X_i,t_* included the predictors of blood demand in the final poisson GEE model, adjusted for changes in ages and body weights.
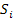
is the annual survival rate for patient *i*.

Imputation of body weights

For the fitting of the GEE model and the prediction of future blood demand from existing patients, we imputed the expected body weight profile, a major predictor of transfusion requirement [2]. More specifically, we adjusted past and future body weights according to the US CDC growth curve [3]. Let *w_a_* be the body weight of an individual at an age of *a* years. *w_a_* was transformed into z-score *z* based on the LMS method [4] as follow:


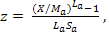


where *L_a_*, *M_a_* and *S_a_* are the power, median and standard deviation for the Box-Cox transformation for those aged *a* years. To obtain the expected weight at a different age *a_2_*, *z* was transformed back to weight *w_a2_* based on parameters *L_a2_, M _a2_* and *S _a2_*.

Prediction of blood demand from new Thalassemia patients

We estimated the number of new cases by assuming a constant age-sex specific incidence rate of new TM patients. Based on the data in 2006-2009 and mid-year population statistics from the Census and Statistics Department [5], we calculated the age-sex specific population incidences for age groups 0-4, 5-9, 10-19 and 20-59 years respectively. The age-sex specific incidences were then applied to the projected population in year 2010-2024.

We predicted the blood demand from these new TM patients by applying the final poisson GEE model, with the mean group age, body weights and expected proportion of TM patients who had undergone splenectomy for each age-sex strata as predictors. For those groups with mean ages <20 years, the mean body weights were imputed in each subsequent year to reflect growth. The blood demand of the new TM patients
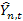
 *t* years after 2009 is given by:


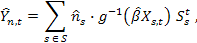


where
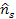
 is the estimated number of new cases in the age-sex strata *s*,
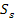
is the annual survival rate for the age-sex strata *s*.

Survival of Thalassemia patients

With better patient management, the survival of TM patients improved in the last decade [6]. To better reflect the expected survival in the near future, we calculated the annual survival rate *S* for TM patients based on the death counts in 2006-2009. In Hong Kong, there was no death in TM patients aged below 20 years during 2006-2009. We calculated the annual death rate for TM patients aged ≥20 years as a discount factor for future blood demand from both existing and new TM patients.

Prediction of total future blood demand for all Thalassemia patients

The total future blood demand
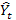
was calculated by the sum of blood demand from existing TM patients and new cases of TM patients, i.e.


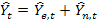


Forecast error of the estimates

Based on the Poisson GEE model and using the robust variance estimator for
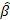
, the forecast error of blood demand
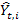
 for patient *i* at year *t* after 2009, before adjusted for survival is given by:


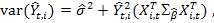


where
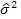
was estimated by *RSS / (N – p)*, and *RSS*, *N* and *p* are the residuals sum of squares, the number of observations and parameters respectively. Assuming independence between survival and blood demand from TM patients, we used the delta method [7] to account for the uncertainty in the estimates of the blood demand, projected new cases of TM patients and survival rate.

**References**

1. Hastie T, Tibshirani R, J F (2001) The Elements of Statistical Learning: Data Mining, Inference, and Prediction. New York: Springer.

2. Thalassemia International Federation (2008) *Guidelines for the Clinical Management of Thalassaemia*. Nicosia: Thalassemia International Federation.

3. Centers for Disease Control and Prevention (2009) Growth Charts. Available: http://www.cdc.gov/growthcharts/data_tables.htm. Accessed 1 October 2011.

4. Cole TJ, Green PJ (1992) Smoothing reference centile curves: the LMS method and penalized likelihood. Stat Med 11: 1305-1319.

5. Census and Statistics Department (2011) Hong Kong Monthly Digest of Statistics, September 2011. Available: http://www.statistics.gov.hk/publication/feature_article/B71109FB2011XXXXB0100.pdf. Accessed 1 November 2011.

6. Modell B, Khan M, Darlison M, Westwood MA, Ingram D, et al. (2008) Improved survival of thalassaemia major in the UK and relation to T2* cardiovascular magnetic resonance. J Cardiovasc Magn Reson 10: 42.

7. Oehlert GW (1992) A note on the delta method. The American Statistician 46: 27-29.
